# Bilateral renal vein thrombosis and pulmonary embolism secondary to membranous glomerulonephritis treated with percutaneous catheter thrombectomy and localized thrombolytic therapy

**DOI:** 10.4103/0971-4065.70848

**Published:** 2010-07

**Authors:** S. P. Janda

**Affiliations:** Department of Medicine, University of British Columbia, Vancouver, British Columbia, Canada

**Keywords:** Membranous glomerulonephritis, pulmonary embolism, renal vein thrombosis

## Abstract

Renal vein thrombosis (RVT) is a rare event but is prevalent in patients with nephrotic syndrome. Bilateral RVT is even rarer. The literature is relatively sparse in terms of the management of RVT because of its rarity and consists of a few case reports and case series. We present a case with bilateral RVT complicated by a pulmonary embolism in a patient with membranous glomerulonephritis (MGN). A 19-year-old female presented with acute flank pain and worsening renal function after a couple of weeks in hospital while being treated with diuretics for anasarca secondary to MGN. Venography was used for diagnosis. The patient underwent percutaneous catheter thrombectomy and localized thrombolysis achieving resolution of pain and improvement of renal function. The patient was then anticoagulated for life with warfarin.

## Introduction

Membranous glomerulonephritis (MGN) is the most common type of the nephrotic syndrome diseases associated with venous thrombosis. The prevalence of renal vein thrombosis (RVT) is between 20-60% in MGN.[1] Pulmonary embolism (PE) is a complication of RVT and deep vein thrombosis (DVT) and prevalence in nephrotic syndrome is estimated to be between 12-30%.[1,2] The clinical presentation of RVT is varied and non-specific. There is no clear consensus for the acute treatment of RVT. Several studies have shown good results with percutaneous catheter thrombectomy and localized thrombolysis.[3–6] Bilateral RVT with PE is less frequent. We present a case of bilateral RVT complicated by PE which was successfully treated with thrombectomy and localized thrombolysis in a patient with recently diagnosed MGN. A review of the literature in terms of presentation and treatment of acute RVT is presented.

## Case Report

A 19-year-old female was admitted to hospital for management of generalized edema secondary to nephrotic syndrome from MGN (stage 1) diagnosed three months ago via a renal biopsy with a 24-hour protein of 5.4 g. Other causes of renal impairment were ruled out via lab tests at that time. Despite being on a regimen of furosemide 160 mg bid and spironolactone 25 mg bid, she gained approximately 25 kg. She was asymptomatic otherwise at the time of admission. Past medical history was unremarkable. On social history, she did not smoke and drank alcohol occasionally. Family history was unremarkable.

On physical examination she was edematous. Vitals were as follows: BP 110/90, pulse 110, RR 20 m, O_2_ saturation 99% on room air. Head and neck examination was unremarkable. Respiratory exam revealed no crackles or wheezes and good breath sounds bilaterally. Cardiovascular examination was unremarkable. She had moderate to severe bilateral pitting edema up into the thighs. Abdominal examination was unremarkable.

Hb 11.5 mg/dL, TLC 8000/mm^3^, platelets 2.65×10^5^/mm^3^, Na 139 mEg/L, K 3.4 mEg/L, urea 11.2 mg/dL, creatinine 0.96 mg/dL, albumin 2.7 mg/dL, ALT 13, AST 16, GGT 14, alk phos 64, LDH 253, bilirubin 0.12 mg/dL. Urinalysis showed the following: Total protein >3.0 gm/day, white blood count (WBC) 11-20, red blood count (RBC) 11-20, many epithelial casts, >10 hyaline casts, 4-10 granular casts, fatty casts, and oval bodies. Chest X-ray was normal.

She was restarted on diuretic therapy but this time at higher doses (furosemide 120 mg IV TID and metalazone 10 mg IV TID). She started losing weight at a rate of 1-2 kg per day.

On day 17 of her admission, she developed sudden onset left sided flank pain that was sharp and localized to the left costovertebral angle. It was 8 to 9 out of 10 in intensity and did not vary with position. She was started on hydromorphone for the pain and an MRI was ordered to rule out renal vein thrombosis. Later that evening, she developed dyspnea and chest pain. Electrocardiography (ECG) showed sinus tachycardia at 116 bpm and an S1Q3T3 pattern. Troponin was mildly elevated at 0.11. She was started on IV heparin for a presumed diagnosis of a PE. A ventilation/perfusion scan was ordered the subsequent morning and revealed a large segmental perfusion defect involving the majority of the right upper lobe as well as the superior segment of the left lower lobe. She continued on her IV heparin and awaited the MRI to rule out RVT as a source of her PE. Doppler ultrasound of her legs was negative for DVT. A hypercoaguability work-up (Factor V Leiden, prothrombin gene mutation 20210, antithrombin III deficiency, protein C and S deficiency, and antiphospholipid antibody) was done and was negative.

**Figure 1:**
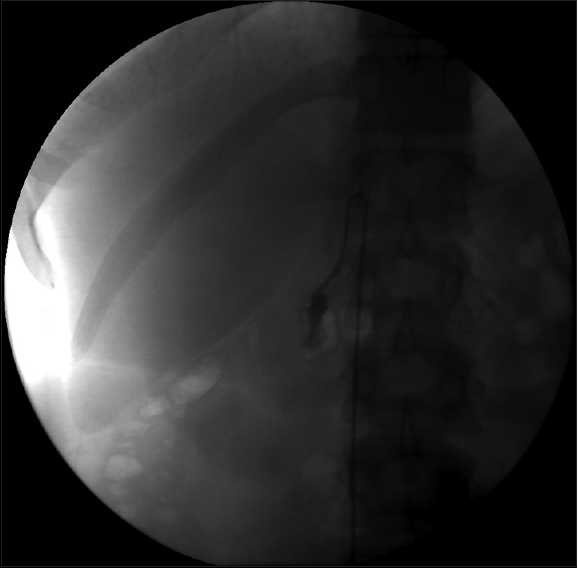
Renal venogram showing the right renal vein with an occlusive or nearly occlusive thrombus in its central aspect with mild extension into the inferior vena cava

**Figure 2:**
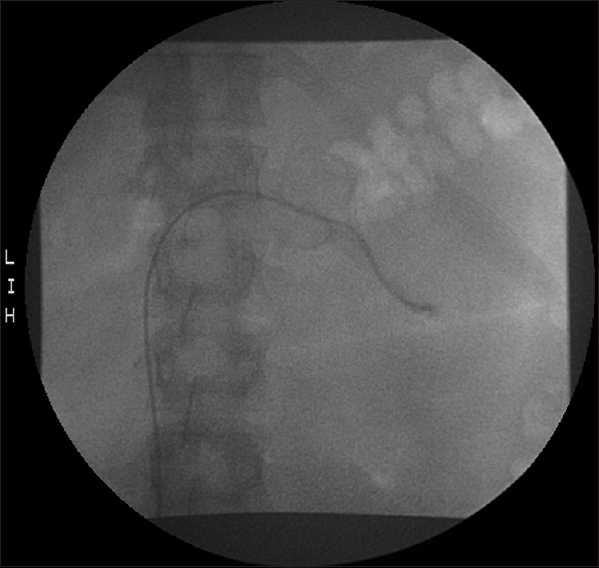
Renal venogram showing the left renal vein with a large occlusive thrombus involving the central 5 cm of the left renal vein with thrombus extending into the inferior vena cava

Her renal function continued to worsen (Cr 1.76 mg/ dL) and as a result her diuretics were held and she was given IV albumin. She could not undergo the MRI because she was claustrophobic and as result, a renal venogram was ordered. On day 19, she underwent a renal venogram which revealed a large occlusive thrombus involving the central 5 cm of the left renal vein with thrombus extending into the inferior vena cava (IVC) [Figure 1]. The right renal vein also contained an occlusive or nearly occlusive thrombus in its central aspect with mild extension into the IVC [Figure 2]. Mechanical thrombolysis of the left renal vein thrombus was carried out with a wire and catheter which were eventually passed beyond the thrombus. An infusion catheter was then placed and thrombolysis with TPA was done. A 1/10 mixture of TPA (0.1 mg/ ml) was mixed and injected at 2 ml/sec for 5 seconds (0.5 mg) every 60 seconds for a total of 24.5 mg of TPA over 49 minutes. Modest improvement was obtained postthrombolytic therapy. It was decided that if renal function continued to worsen, repeat thrombolysis be done in a couple of days. Her renal function continued to worsen (Cr 2.08) and thrombolysis with TPA was performed again, this time on the right, on day 21 for total dose of 20 mg of TPA over 40 minutes.

Her renal function subsequently improved and she was back to a baseline creatinine of 110 by day 29. She was started on mycophenolate mofetil 1,000 mg bid for treatment of her MGN. It was determined that she required life-long anticoagulation with coumadin with an INR goal between the range of 2-3. She continued her diuretics (furosemide 80 mg bid and spironolactone 100 mg daily) to maintain a goal weight of 130 kg. She was discharged home on day 29.

## Discussion

RVT is a complication of nephrotic syndrome. MGN is the most common cause of nephrotic syndrome that is associated with venous thrombosis followed by membranoproliferative glomerulonephritis and then minimal change disease.[1,7]

The mechanism of the hypercoaguable state leading to venous thrombosis in nephrotic syndrome is poorly understood. One study looked at hemostatic molecular markers in nephrotic syndrome and found that compared to controls, individuals with nephrotic syndrome had higher levels of fibrinopeptide A and lower levels of thrombin-antithrombin complex (due to urinary losses) concluding intravascular hemostatic activation in nephrotic syndrome.[8] Another study suggests that hemoconcentration by glomeruli, which is further exacerbated by diuretic therapy in nephrotic syndrome, predisposes to RVT.[9]

The clinical presentation of RVT is variable. It can present unilateral, bilateral, and may have extension into the IVC. Acute presentation of RVT is less common in nephrotic syndrome and usually presents with flank pain, microscopic or gross hematuria, increase in renal size on radiographic exams, and renal failure if RVT is bilateral.[10] Chronic RVT is more common in nephrotic syndrome and usually is asymptomatic and is often discovered after a PE or DVT.[1,11,12]

Diagnosis of RVT can be made by various modalities including CT, MRI, duplex ultrasonography, intravenous pyelography, and venography. Venography is the gold standard for diagnosis of RVT but noninvasive modalities such as CT with contrast or MRI have been shown to be good alternatives in several studies.[13,14] Radiographic signs of RVT include the following: Increased renal size; enlarged renal vein; delayed, diminished, or absent opacification of the collecting system; prolonged corticomedullary differentiation; thickening of renal fascia; and stranding of perinephric fat.[15,16] Acute RVT usually presents with a visible clot in an enlarged renal vein, whereas chronic RVT presents with renal vein retraction with collateral vessel prominence.[17]

Treatment of RVT is aimed at restoration of renal function. Treatment includes anticoagulation, thrombolysis, and surgery. In those individuals with asymptomatic RVT, there are no randomized controlled trials or observational trials that evaluate the role of aniticogulation. A few case series have found some benefit.[1,11] Symptomatic RVT is treated with anticoagulation, initially with heparin and then with warfarin with an INR target of 2.0-3.0.[18] The duration of anticoagulation is uncertain but most experts recommend at least one year up to lifetime.[10,19] Localized thrombolysis with catheter thrombectomy for acute RVT has proven to be a successful treatment.[3–6] One retrospective review looked at acute RVT treated with percutaneous catheter-directed thrombectomy with or without thrombolysis. Seven thrombosed renal veins in six patients were treated with thrombectomy and five renal veins were additionally treated with thrombolysis for mean duration of 22 hours. Restoration of flow to the renal veins was achieved in all thrombosed veins and there was both clinical improvement as well as renal function improvement.[4] Surgical thrombectomy is only recommended for patients with bilateral RVT with renal impairment who cannot undergo a percuteneous procedure.[20]

The current case illustrates an acute presentation of RVT in a patient with MGN. Clinical presentation with acute flank pain was consistent with acute RVT versus chronic RVT. Bilateral RVT was suggested by worsening renal function. Because the patient could not tolerate a MRI, was too large to get a good quality duplex ultrasound, and had worsening renal function to consider CT with contrast, the gold standard venography was done to obtain the diagnosis as well as therapeutic intervention. There was modest improvement in renal flow and complete recovery of renal function back to baseline with catheter thrombectomy and localized thrombolysis consistent with previous studies.[3–6]
